# Molecular Mechanism of Indoor Exposure to Airborne Halogenated Flame Retardants TCIPP (Tris(1,3-Dichloro-2-Propyl) Phosphate) and TCEP Tris(2-chloroethyl) Phosphate and Their Hazardous Effects on Biological Systems

**DOI:** 10.3390/metabo14120697

**Published:** 2024-12-10

**Authors:** Albatul Alharbi, Muhanad Alhujaily

**Affiliations:** Department of Clinical Laboratory Sciences, College of Applied Medical Sciences, University of Bisha, Bisha 61922, Saudi Arabia; alalharbi@ub.edu.sa

**Keywords:** airborne halogenated flame retardants, TCIPP (tris(1,3-dichloro-2-propyl) phosphate), TCEP (tris(2-chloroethyl) phosphate), flame retardants, human exposure, indoor air quality

## Abstract

TCIPP (tris(1,3-dichloro-2-propyl) phosphate) and TCEP (tris(2-chloroethyl) phosphate) are organophosphate ester flame retardants found in various consumer products, posing significant health and environmental risks through inhalation, ingestion, and dermal exposure. Research reveals these compounds cause oxidative stress, inflammation, endocrine disruption, genotoxicity, neurotoxicity, and potentially hepatotoxicity, nephrotoxicity, cardiotoxicity, developmental, reproductive, and immunotoxicity. This review summarizes the current knowledge on the toxicological mechanisms of TCIPP and TCEP and presents the latest data on their toxicological effects obtained in vitro and in vivo, using omic systems, and on the basis of computational modelling. It also elaborates on the scope of further toxicities and highlights the necessity of ongoing mechanistic research, integration of new technologies, and successful transfer of the acquired knowledge into risk evaluation, policies and regulations, and the creation of safer products. Since flame retardants are already present in homes, schools, offices, and daycare centres, efforts to scale back the exposure to these chemicals, most especially the hazardous ones, must be made to protect human health and the environment. Therefore, effective and timely prevention, based upon a deep knowledge of the entire toxicological profile of these substances, is the only way to face this difficult toxicological issue and provide for a healthy and safe future.

## 1. Introduction

TCIPP and TCEP are two common flame retardants used in a wide variety of products including furniture, textiles, electronics, and building materials [1]. These organophosphate esters are used in applications increasing the fire resistance of materials by suppressing the combustion process. Nonetheless, they have been shown to be toxic in most uses and thus, the need to determine their toxic effects on the health of humans and the environment [2].

TCIPP is composed of a phosphate group with three 1,3-dichloro-2-propyl groups bound to the phosphorus atom, which has chemical formula C_9_H_15_Cl_6_O_4_P and a molecular mass of 430.9 g/mol. It is a non-hygroscopic liquid, which is colourless to pale yellow with a mild odour, a boiling point of 237 °C and a low vapour pressure [3]. On the other hand, TCEP contains a phosphate group with three 2-chloroethyl groups, chemical formula C_6_H_12_Cl_3_O_4_P and a molecular mass of 285.5 g/mol [4]. TCEP is a non-hazardous, colorless liquid with a low boiling point of 148 °C and is less flammable, as compared to TCIPP [5]. Flame retardants both act as fire retardants and are compatible with different polymers and resins used in the production of these materials. They are widely integrated into polyurethane foams, textiles, and other products applied in furniture production to conform to fire requirements, and people can be exposed to them through skin contact, inhalation of dust particles, or from the off-gassing of the products [6]. They are also employed in insulating materials, construction foams, and other building elements; people can be exposed to them through installation or demolition of the substances, as well as through the emission of compounds into indoor air. Flame retardants are added to electronic equipment and products to prevent fire risks in computers, TVs, and circuit boards among other products and people can be exposed to these chemicals during manufacturing, usage, and even during the disposal of electronic products. In the automotive industry, they are found in car interiors, insulating materials and electrical components, and exposure may be during automobile manufacturing, usage or repair work [7].

There are three main routes of exposure to TCIPP and TCEP: inhalation of dust or vapours, ingestion of contaminated food or water and dermal absorption through skin contacts with products containing these compounds and environment exposure through release of these compounds in air, water or soil and accumulation in food chains [8]. It is important to better understand the molecular toxic effects of TCIPP and TCEP since these compounds have been identified in maternal blood, breast milk, and urine and other biological matrices of people from various countries. Understanding of these mechanisms is essential for evaluating possible impact on human health, including cancer, reproductive and developmental toxicity, endocrine disruption, neurotoxicity and for identification of biomarkers for exposure and effect, refinement of risk assessment models and approaches to the design of targeted therapeutic and preventive strategies. These compounds are chemically stable and have been reported in different media; air, water, soil and biota and therefore their possible effects on ecosystems have to be assessed to aid in the process of cleaning up [9]. This understanding informs environmental monitoring and management strategies to mitigate the release and spread of these compounds and aids in the development of effective remediation technologies. Regulatory decision-making benefits from this knowledge, as it helps establish appropriate exposure limits, risk assessments, and guidelines for the use and disposal of products containing TCIPP and TCEP, facilitating evidence-based regulations and policies to protect public health and the environment. It also guides the evaluation of potential alternatives or substitutes for TCIPP and TCEP, ensuring safer options are explored and adopted.

By elucidating the specific molecular pathways involved in their toxicity, researchers can develop safer alternatives or substitutes that do not exhibit similar toxic effects, driving the development of more sustainable and environmentally friendly flame retardants, contributing to a circular economy, and promoting green chemistry principles. This mechanistic understanding enables the design and synthesis of new flame retardants with improved safety profiles and aids in developing effective treatments for individuals exposed to these compounds, mitigating potential health consequences and improving public health outcomes. Investigating the molecular mechanisms of toxicity also provides insights into the fundamental processes involved in toxicity, such as oxidative stress, DNA damage, endocrine disruption, and neurological effects, contributing to the broader field of toxicology and advancing our knowledge of how environmental contaminants interact with biological systems at the molecular level. It reveals potential similarities or differences in the mechanisms of action between TCIPP and TCEP, informing risk assessment and management strategies [10].

To elucidate the molecular mechanisms of toxicity, researchers employ a range of experimental approaches, including in vitro and in vivo studies, omics technologies (e.g., transcriptomics, proteomics, metabolomics), and computational modelling [11]. These techniques allow for the examination of cellular and molecular processes, such as gene expression changes, protein modifications, metabolic alterations, and interactions with biological targets [12]. In vitro studies using cell culture models provide insights into the direct effects of TCIPP and TCEP on various cell types, including those relevant to potential target organs or systems. These studies can investigate mechanisms such as oxidative stress, DNA damage, endocrine disruption, and cytotoxicity. Additionally, advanced techniques like high-throughput screening and computational modelling can aid in identifying potential molecular targets and elucidating structure-activity relationships [13]. In vivo studies using animal models, such as rodents or zebrafish, allow for the assessment of systemic effects and the investigation of toxicokinetics (absorption, distribution, metabolism, and excretion). These studies can provide valuable information on the potential adverse effects of TCIPP and TCEP on various organs and systems, including reproductive, developmental, neurological, and carcinogenic effects [14,15,16]. Omics technologies, including transcriptomics (gene expression analysis), proteomics (protein expression and modification analysis), and metabolomics (metabolite profiling), can provide invaluable insights into the molecular mechanisms of toxicity. These techniques enable researchers to comprehensively analyze the cellular responses to TCIPP and TCEP exposure, identifying altered gene expression patterns, protein modifications, and metabolic perturbations that may contribute to toxicity [17,18,19]. Transcriptomic studies can reveal changes in gene expression profiles associated with various toxicity pathways, such as oxidative stress, inflammation, DNA damage response, and endocrine disruption [20]. These data can help identify potential biomarkers of exposure and effect, as well as elucidate the underlying molecular mechanisms. Proteomic analyses can detect changes in protein abundance, post-translational modifications, and protein-protein interactions in response to TCIPP and TCEP exposure. These alterations can provide insights into the disruption of cellular signalling pathways, enzyme activities, and structural proteins, which may contribute to the observed toxic effects [21].

Metabolomic studies can identify changes in metabolite levels and metabolic pathways that are perturbed by TCIPP and TCEP exposure. These changes can shed light on the potential mechanisms of toxicity, such as oxidative stress, disruption of energy metabolism, and alterations in lipid, amino acid, or nucleic acid metabolism.

By integrating data from these omics technologies, researchers can gain a comprehensive understanding of the molecular events underlying the toxic effects of TCIPP and TCEP, including the identification of key molecular targets, pathways, and potential biomarkers. Computational modelling approaches, such as molecular docking, quantitative structure-activity relationship (QSAR) modelling, and systems biology models, can complement experimental studies [22]. These techniques can aid in predicting potential interactions between TCIPP and TCEP with biological targets, identifying structural features that contribute to toxicity, and simulating the complex networks of molecular interactions involved in toxicity pathways. Furthermore, advanced analytical techniques, such as high-resolution mass spectrometry and nuclear magnetic resonance (NMR) spectroscopy, can provide valuable information on the metabolic fate and biotransformation of TCIPP and TCEP within biological systems [23]. Understanding the formation of reactive metabolites and their potential interactions with cellular components can shed light on the mechanisms of toxicity. Elucidating the molecular mechanisms of toxicity for TCIPP and TCEP is a multidisciplinary endeavor that requires the integration of various experimental and computational approaches. By combining data from in vitro and in vivo studies, omics technologies, and computational modelling, researchers can gain a comprehensive understanding of the cellular and molecular events underlying the toxic effects of these flame retardants. This knowledge can inform risk assessment strategies, guide the development of safer alternatives, and contribute to the protection of human health and the environment. Additionally, mechanistic insights can aid in the identification of potential biomarkers for exposure and effect monitoring, as well as inform the development of targeted therapeutic interventions or preventive measures.

It is important to note that while significant progress has been made in elucidating the molecular mechanisms of toxicity for TCIPP and TCEP, there are still knowledge gaps and areas that require further investigation. Continued research efforts, involving interdisciplinary collaborations and the application of cutting-edge technologies, will be essential to fully unravel the complex mechanisms underlying the toxic effects of these widely used flame retardants.

## 2. Metabolism and Biotransformation

**Figure 1:**
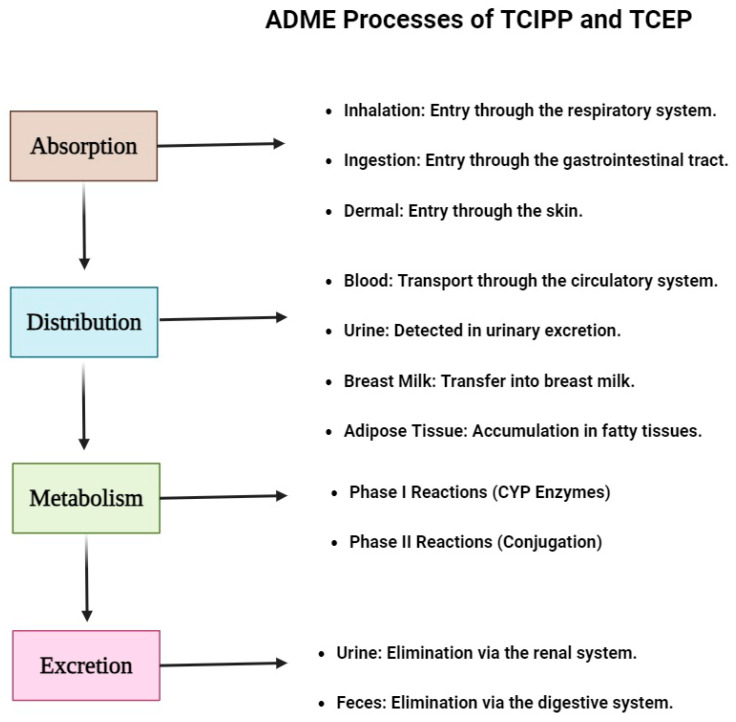
The processes of the flame retardants TCIPP and TCEP in the human body are depicted in this diagram: absorption, distribution, metabolism, and excretion. It outlines the main pathways for absorption (ingestion, inhalation, and dermal absorption), the distribution of the substance to different tissues and bodily fluids (blood, urine, breast milk, and adipose tissue), the metabolic pathways involving Phase I and Phase II reactions, and the pathways for excretion (urine and feces). By giving a thorough description of how these substances are metabolized within the body, the diagram aims to advance knowledge of their toxicokinetic characteristics and potential health hazards.

The absorption, distribution, metabolism, and excretion (ADME) of TCIPP and TCEP play a crucial role in determining their toxicokinetic profiles and potential toxic effects within the human body [24]. Knowledge of these processes is crucial for the evaluation of potential dangers connected with the contact with these flame retardants and for the description of their molecular toxic effects. Inhalation, ingestion, and dermal penetration are some of the methods of exposure to TCIIP and TCEP that can be absorbed into the body [25] (Figure 1). Inhalation is thought to be a major route of exposure, especially in the workplace or other enclosed environments that contain these substances in the form of dust or vapors (Figure 1). Some investigations have demonstrated that both TCIPP and TCEP can penetrate the lungs and be transported into the bloodstream. Characteristically, TCIPP and TCEP can be ingested through contaminated food and water or through the inhalation of contaminated dust; these compounds can be absorbed through the gastrointestinal tract due to their lipophilic properties, which enables their passage through the intestinal epithelium and into the bloodstream [26]. Another possible route of exposure is skin, which can occur in occupational conditions or when using products containing TCIPP and TCEP, although the degree of skin penetration depends on the concentration, formulation, and duration of exposure [27]. After absorption, TCIPP and TCEP can travel through the systemic circulation and the distribution and accumulation of these species in tissues can be affected by several factors such as lipophilicity, protein binding and metabolism (Figure 1). Published research has identified TCIPP and TCEP in different human body tissues and in biological fluids like blood, urine, breast milk, and adipose tissue, which indicates that these compounds can reach different organs and tissues with relative ease, where their lipophilic character may allow for their storage in adipose tissues and thus cause long-term exposure and toxicity [28]. TCIPP and TCEP undergo metabolic biotransformation catalyzed by various enzymes, primarily in the liver but also in other tissues, involving Phase I and Phase II reactions that can lead to the formation of metabolites with varying toxicological properties [29]. Phase I reactions involve cytochrome P450 (CYP) enzymes that catalyze oxidative reactions such as hydroxylation, dehalogenation, and dichlorination of TCIPP and TCEP, with different CYP isoforms, including CYP3A4, CYP2B6, and CYP2E1, implicated in their metabolism. Phase II reactions involve conjugation with endogenous molecules, such as glucuronic acid or sulfate, catalyzed by enzymes like UDP-glucuronosyltransferases (UGTs) and sulfotransferases (SULTs), resulting in more water-soluble metabolites that facilitate their excretion [30]. Additionally, glutathione S-transferases (GSTs) catalyze the conjugation of TCIPP and TCEP, or their metabolites, with the tripeptide glutathione, facilitating the elimination of reactive metabolites or contributing to the formation of reactive species that can lead to toxicity. The metabolic biotransformation of TCIPP and TCEP can lead to the formation of reactive metabolites that contribute to their toxic effects through various mechanisms, including oxidative stress, covalent binding, glutathione depletion, endocrine disruption, mitochondrial dysfunction, and immunotoxicity. Certain metabolites may undergo redox cycling or interact with cellular components, leading to the generation of reactive oxygen species (ROS) that cause oxidative damage to DNA, proteins, and lipids, contributing to genotoxicity, cellular dysfunction, and inflammation. Reactive metabolites containing electrophilic centers or leaving groups can form covalent adducts with nucleic acids, proteins, or other cellular macromolecules, disrupting normal cellular functions, interfering with enzyme activities, or inducing DNA damage, potentially leading to mutagenicity and carcinogenicity [31]. The conjugation of TCIPP and TCEP metabolites with glutathione can deplete cellular glutathione levels, compromising the antioxidant defense system and increasing susceptibility to oxidative stress and toxicity. Some metabolites may interact with hormone receptors or disrupt hormonal signaling pathways, leading to endocrine disruption and potential adverse effects on reproductive health, development, and metabolic processes [32]. Certain reactive metabolites may accumulate in mitochondria and interfere with their functions, disrupting energy metabolism, increasing ROS production, and causing potential mitochondrial toxicity. Reactive metabolites or their adducts with cellular components may trigger immune responses, leading to inflammation, autoimmune reactions, or immunosuppression. The formation and toxicity of reactive metabolites can be influenced by factors such as genetic variability in metabolic enzymes, co-exposure to other substances that modulate enzyme activities, and individual susceptibility factors like age, sex, and health status [33]. The excretion of TCIPP, TCEP, and their metabolites occurs primarily through urine and, to a lesser extent, feces, with the rate of excretion influenced by factors such as the extent of metabolic biotransformation, protein binding, and individual variability in renal and hepatic function. Studies have detected TCIPP and TCEP metabolites, including their hydroxylated and conjugated forms, in human urine samples, indicating that urinary excretion is a significant route of elimination for these compounds and their metabolites [34].

It is important to note that while the ADME processes and metabolic biotransformation of TCIPP and TCEP have been studied, there are still knowledge gaps and uncertainties regarding the precise metabolic pathways, the formation and toxicological properties of specific metabolites, and the interindividual variability in metabolic profiles. (Table 1) Ongoing research efforts utilizing advanced analytical techniques, such as metabolomics and high-resolution mass spectrometry, can provide further insights into the complex metabolic fate and toxicokinetic of these flame retardants [29]. Understanding the ADME processes and the role of metabolic enzymes in the biotransformation of TCIPP and TCEP is crucial for assessing their toxic potential, identifying potential biomarkers of exposure and effect, and developing effective risk assessment and management strategies. (Table 1) Additionally, this knowledge can guide the development of safer alternatives or targeted interventions to mitigate the adverse effects associated with exposure to these flame retardants.

## 3. Oxidative Stress and Inflammatory Responses

Oxidative stress and inflammatory responses are key molecular mechanisms contributing to the toxicity of TCIPP and TCEP. These processes can lead to cellular damage, tissue injury, and potential adverse health effects. TCIPP and TCEP have been shown to induce oxidative stress in various experimental models, including cell culture systems and animal studies [1,35]. Oxidative stress occurs when there is an imbalance between the production of reactive oxygen species (ROS) and the cellular antioxidant defense mechanisms, leading to an accumulation of oxidants [36]. Some studies have suggested that TCIPP and TCEP, or their metabolites, can directly generate ROS through redox cycling or interactions with cellular components, including superoxide radicals, hydrogen peroxide, and hydroxyl radicals (Table 1). They can also indirectly contribute to ROS generation by disrupting cellular processes involved in redox homeostasis, such as mitochondrial dysfunction, interference with the mitochondrial electron transport chain and ATP production, leading to increased leakage of electrons and ROS generation [37]. Additionally, the conjugation of TCIPP and TCEP metabolites with glutathione can deplete cellular antioxidant reserves, reducing the ability to neutralize ROS, and upregulating enzymes like NADPH oxidases, which generate ROS as part of their catalytic activity [38]. The generated ROS can interact with and damage various cellular components, leading to oxidative stress-mediated toxicity, such as DNA damage through direct oxidation of DNA bases or inducing DNA strand breaks, potentially leading to mutagenesis and carcinogenesis. ROS can initiate lipid peroxidation reactions, particularly in cell membranes, causing structural and functional alterations and potentially leading to cell death. They can also oxidize amino acid residues in proteins, leading to changes in protein structure and function, enzyme inactivation, and potential interference with cellular signaling pathways (Table 2). Excessive ROS can disrupt redox-sensitive signaling pathways involved in cell proliferation, differentiation, and survival, leading to cellular dysfunction and potential tissue damage [39]. Oxidative stress induced by TCIPP and TCEP can trigger inflammatory responses by activating various signaling pathways and transcription factors involved in the regulation of pro-inflammatory mediators. ROS can activate the nuclear factor-kappa B (NF-κB) pathway, a master regulator of inflammation, leading to the expression of various pro-inflammatory cytokines, chemokines, and enzymes [40]. Studies have shown that exposure to TCIPP and TCEP can induce the production of pro-inflammatory cytokines, such as interleukin-6 (IL-6), tumor necrosis factor-alpha (TNF-α), and interleukin-1 beta (IL-1β), in various cell types. Mitogen-activated protein kinase (MAPK) signaling cascades, including p38 MAPK and JNK, can be activated by oxidative stress and participate in the regulation of inflammatory responses [41]. Additionally, TCIPP and TCEP exposure has been associated with increased expression of cyclooxygenase-2 (COX-2), an enzyme involved in the synthesis of pro-inflammatory prostaglandins [42]. The activation of inflammatory pathways can lead to the release of various pro-inflammatory mediators, contributing to tissue injury and systemic effects. Pro-inflammatory cytokines, such as IL-6, TNF-α, and IL-1β, can promote inflammatory responses, induce acute phase proteins, and contribute to the recruitment of immune cells to sites of inflammation. Chemokines, like IL-8 and MCP-1, are involved in the chemotaxis and recruitment of immune cells, such as neutrophils and macrophages, to sites of inflammation. Increased production of prostaglandins, mediated by COX-2 induction, can contribute to inflammation, fever, and pain responses [43,44]. Inflammation can also lead to the generation of reactive nitrogen species, such as nitric oxide and peroxynitrite, which can further exacerbate oxidative stress and cellular damage [45]. The oxidative stress and inflammatory responses induced by TCIPP and TCEP can contribute to various toxic effects and pathological conditions, such as genotoxicity and carcinogenesis due to oxidative DNA damage and inflammation-mediated genomic instability, increasing the risk of mutagenesis and carcinogenesis [46] (Table 2). Oxidative stress and inflammation can disrupt normal cellular processes involved in reproductive function and embryonic development, potentially leading to adverse reproductive and developmental outcomes [47]. Neurotoxicity can result from oxidative stress and neuroinflammation, contributing to neuronal damage, impaired cognitive function, and neurodegenerative processes [48]. Chronic inflammation and oxidative stress have been implicated in the development of metabolic disorders, such as insulin resistance, obesity, and dyslipidemia. Oxidative stress and inflammation can promote endothelial dysfunction, atherosclerosis, and cardiovascular disease. Excessive ROS production and inflammatory responses in the liver and kidneys can lead to tissue injury and organ dysfunction [49] (Table 2). The severity and specific manifestations of oxidative stress and inflammatory responses induced by TCIPP and TCEP can vary depending on factors such as exposure duration, dose, individual susceptibility, and the presence of other environmental stressors or co-exposures [50]. Ongoing research efforts are focused on further elucidating the molecular mechanisms underlying the oxidative stress and inflammatory responses induced by these flame retardants, as well as identifying potential biomarkers and therapeutic interventions to mitigate their adverse effects. Additionally, the development of safer alternatives with reduced oxidative and inflammatory potential is a priority in the pursuit of more sustainable and environmentally friendly flame retardants.

## 4. Endocrine Disruption

**Figure 2:**
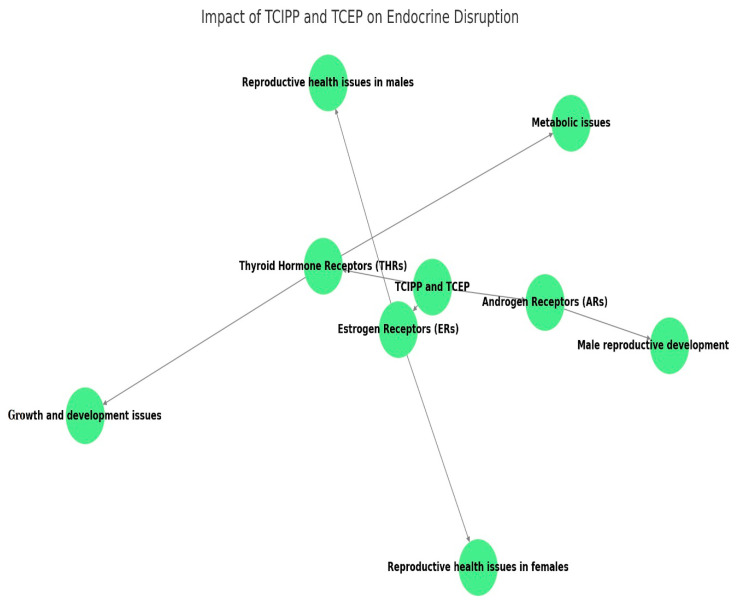
For endocrine disruption, TCEP and TCIPP effects are shown in Figure 2. It highlights the procedure of interaction between these compounds and three central hormone receptors such as thyroid hormone receptors (THRs), androgen receptors (ARs) and estrogen receptors (ERs). The result is a summary of the main outcomes of these interactions which shows that alteration to ERs may lead to fertility problems in both men and women, changes to ARs lead to male reproductive defects, while alterations in THRs have growth defects and metabolic disorders.

Endocrine disruption is a growing concern associated with exposure to various environmental contaminants, including flame retardants such as TCIPP and TCEP. These compounds have been shown to interfere with hormone receptors and signaling pathways, potentially leading to adverse effects on reproductive health, development, and metabolic processes [51,52] (Figure 2). Several studies have demonstrated that TCIPP and TCEP can interact with estrogen receptors (ERs), which are crucial for regulating various physiological processes, including reproductive function, bone health, and the cardiovascular system [53,54].

These compounds have been shown to exhibit both estrogenic and anti-estrogenic activities, depending on the experimental conditions and concentrations, and can bind to ERs and modulate their transcriptional activity, leading to changes in the expression of estrogen-responsive genes [54]. Evidence suggests that TCIPP and TCEP can also interact with androgen receptors (ARs), essential for male sexual development, reproductive function, and various metabolic processes. These compounds have been reported to exhibit anti-androgenic activities, potentially disrupting androgen-mediated signalling pathways and gene expression [55]. Additionally, TCIPP and TCEP have been implicated in disrupting thyroid hormone homeostasis, which is crucial for proper growth, development, and metabolic regulation. These compounds can interfere with the synthesis, transport, and metabolism of thyroid hormones, leading to alterations in their circulating levels and disrupting their signaling pathways (Table 3) Potential mechanisms include inhibition of thyroid hormone-metabolizing enzymes, disruption of thyroid hormone transport proteins, and modulation of thyroid hormone receptor activity [56]. Studies have also suggested that TCIPP and TCEP may interact with other nuclear receptors, such as the peroxisome proliferator-activated receptors (PPARs), involved in lipid and glucose metabolism, as well as inflammatory responses. These interactions can potentially disrupt metabolic homeostasis and contribute to the development of metabolic disorders [57]. The interference of TCIPP and TCEP with hormone receptors and signaling pathways can lead to disruptions in estrogen, androgen, and thyroid hormone homeostasis, resulting in various adverse effects. Disruption of estrogen and androgen signalling can affect reproductive function in both males and females. In females, alterations in estrogen levels and signaling may lead to disruptions in ovarian function, menstrual cycle irregularities, and potential fertility issues. In males, anti-androgenic effects can impair spermatogenesis, sperm quality, and male reproductive organ development and function. Exposure to endocrine-disrupting compounds like TCIPP and TCEP during critical developmental windows can have profound effects on growth, development, and organogenesis. Disruptions in estrogen, androgen, and thyroid hormone signalling can interfere with proper tissue and organ development, potentially leading to congenital malformations, neurodevelopmental deficits, and metabolic programming [56,58]. Thyroid hormones play a crucial role in regulating metabolic processes, including energy homeostasis, lipid and glucose metabolism, and thermogenesis [59]. Disruptions in thyroid hormone homeostasis induced by TCIPP and TCEP can contribute to the development of metabolic disorders such as obesity, insulin resistance, and dyslipidemia [60,61]. Interactions with other nuclear receptors, such as PPARs, can further exacerbate metabolic dysregulation and increase the risk of metabolic syndrome (Table 3) The endocrine-disrupting properties of TCIPP and TCEP have raised concerns about their potential impacts on human health, particularly in vulnerable populations. Exposure to these compounds during critical windows of development or adulthood may contribute to reproductive disorders, such as infertility, endometriosis, and polycystic ovary syndrome (PCOS) in females, and impaired spermatogenesis and reduced sperm quality in males [54,62]. Prenatal or early life exposure to endocrine disruptors like TCIPP and TCEP can potentially lead to neurodevelopmental deficits, congenital malformations, and increased susceptibility to metabolic disorders later in life. Disruption of thyroid hormone homeostasis and interactions with nuclear receptors involved in metabolic regulation may contribute to the development of obesity, type 2 diabetes, dyslipidemia, and other metabolic disorders. (Table 3) Emerging evidence suggests that exposure to endocrine disruptors can have transgenerational effects, potentially leading to adverse health outcomes in subsequent generations through epigenetic mechanisms [63,64,65].

The endocrine-disrupting effects of TCIPP and TCEP can be influenced by various factors, including exposure levels, timing, and duration, as well as individual susceptibilities and co-exposures to other environmental contaminants [66]. Ongoing research efforts are focused on further investigating the molecular mechanisms underlying the endocrine-disrupting properties of these flame retardants, as well as assessing their potential impacts on human health and identifying susceptible populations. This knowledge is crucial for informing regulatory decisions, risk assessment, and the development of safer alternatives to mitigate the potential adverse effects associated with exposure to these compounds. Additionally, epidemiological studies and biomonitoring efforts are essential for evaluating the real-world exposure levels and potential health outcomes associated with TCIPP and TCEP exposure in human populations. By integrating data from various research approaches, a comprehensive understanding of the endocrine-disrupting effects of these flame retardants can be achieved, ultimately contributing to the protection of human health and the development of effective risk management strategies.

## 5. Genotoxicity and Carcinogenicity

**Figure 3:**
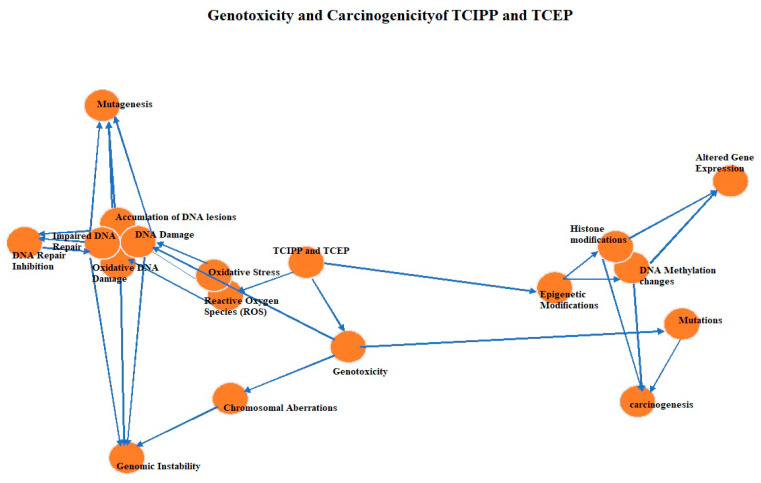
Genotoxic and carcinogenic mechanisms of TCIPP and TCEP. This figure shows genotoxicity, oxidative stress, inhibition of DNA repair, and Epigenetic Modifications as the four major pathways. The different types of DNA damage and cellular effects, such as mutations, chromosomal aberrations, oxidative DNA damage, and impaired DNA repair, are caused by these pathways. They can lead to mutagenesis, genomic instability, altered gene expression, and ultimately carcinogenesis.

The evaluation of the genotoxic and carcinogenic potential of TCIPP and TCEP is crucial for assessing the potential health risks associated with exposure to these flame retardants. These compounds have been extensively studied for their ability to induce DNA damage and their potential to initiate or promote carcinogenesis [5,13] (Figure 3).

Genotoxicity, which refers to the ability of a substance to damage genetic material, including DNA and chromosomes, can lead to mutations and potentially contribute to the development of cancer or other genetic disorders [67]. In vitro genotoxicity studies, such as bacterial mutagenicity assays (Ames Test), have generally shown negative or weakly positive results for TCIPP and TCEP, indicating limited gene mutation potential in bacterial strains [5,68,69,70]. However, mammalian cell genotoxicity assays have provided mixed results, with some studies reporting positive findings for chromosomal aberrations, micronucleus formation, and DNA strand breaks induced by these compounds [71,72]. Micronucleus assays, which detect chromosomal damage and aneuploidy, have reported positive findings in some studies and negative or equivocal outcomes in others [73]. Comet assays, which detect DNA strand breaks, have shown positive results for both TCIPP and TCEP in various experimental models, suggesting their potential to induce DNA damage [13,74].

The mechanisms by which TCIPP and TCEP can potentially induce DNA damage involve both direct and indirect pathways. These compounds have been shown to induce oxidative stress through the generation of reactive oxygen species (ROS), leading to oxidative DNA damage (Table 4) ROS can cause various types of DNA lesions, including oxidized bases, a basic sites, and single- and double-strand breaks, which can contribute to mutagenesis and genomic instability [5,74,75]. Furthermore, TCIPP and TCEP have been reported to inhibit enzymes involved in DNA repair mechanisms, such as DNA glycosylases and topoisomerases, which play crucial roles in maintaining genomic integrity [5]. Impaired DNA repair can lead to the accumulation of unrepaired DNA lesions, increasing the risk of mutagenesis and carcinogenesis [76]. Emerging evidence also suggests that TCIPP and TCEP may induce epigenetic modifications, such as DNA methylation changes and histone modifications, which can alter gene expression patterns and potentially contribute to carcinogenesis [71]. These epigenetic effects can be transmitted to subsequent generations, potentially leading to transgenerational effects on genomic stability and cancer risk [77]. Based on the available evidence, TCIPP and TCEP have been classified as potential carcinogens by various regulatory agencies [27]. The International Agency for Research on Cancer (IARC) has classified TCIPP as Group 3 (“Not classifiable as to its carcinogenicity to humans”) due to limited evidence in experimental animals and inadequate evidence in humans [78], while TCEP is classified as Group 2A (“Probably carcinogenic to humans”) based on sufficient evidence of carcinogenicity in experimental animals [13]. The potential carcinogenic mechanisms of TCIPP and TCEP may involve both initiating and promoting events in the multistep process of carcinogenesis. Induction of DNA damage, including mutations and chromosomal aberrations, can initiate the carcinogenic process by altering the genetic material of normal cells (Table 4). Oxidative stress and direct interactions with DNA can contribute to the accumulation of genetic lesions, increasing the risk of cellular transformation and the development of precancerous lesions. Additionally, TCIPP and TCEP may act as tumor promoters by inducing cellular proliferation, inhibiting apoptosis (programmed cell death), and creating a pro-inflammatory environment that supports the growth and survival of initiated cells [74]. It is important to note that the carcinogenic potential of TCIPP and TCEP may be influenced by various factors, including exposure levels, duration, and individual susceptibilities. Additionally, co-exposure to other environmental carcinogens or lifestyle factors may further modulate the carcinogenic risk associated with these flame retardants.

Ongoing research is focused on investigating the genotoxic and carcinogenic mechanisms of TCIPP and TCEP, as well as evaluating their potential to induce specific cancers in experimental models and epidemiological studies. This knowledge is crucial for informing risk assessments and regulatory decisions regarding these flame retardants. Developing safer alternatives with reduced genotoxic and carcinogenic potential is a priority for more sustainable and environmentally friendly solutions. By integrating data from in vitro and in vivo studies, omics technologies, and computational modeling, a comprehensive understanding of TCIPP and TCEP’s genotoxic and carcinogenic potential can be achieved. This will ultimately contribute to protecting human health and developing effective risk management strategies.

## 6. Neurotoxicity

TCIPP and TCEP, widely used as flame retardants, have raised significant concerns about their potential neurotoxic effects on the central and peripheral nervous systems [1]. Accumulating evidence from various studies suggests that exposure to these compounds can adversely impact neurological functions through a range of mechanisms, including altered neurotransmitter levels, oxidative stress, and disruption of neural signaling pathways [79]. (Table 5) In the central nervous system (CNS), animal studies have demonstrated that exposure to TCIPP and TCEP can lead to neurobehavioral changes such as altered locomotor activity, impaired learning and memory, and changes in anxiety-like and depressive-like behaviors [80]. These behavioral alterations may be linked to disruptions in neuronal circuits involved in cognitive functions, emotional regulation, and motor coordination. Moreover, exposure to TCIPP and TCEP during critical developmental windows, such as prenatal and early postnatal periods, has been linked to neurodevelopmental deficits in animal models. These include impaired brain growth, altered neuronal migration and differentiation, and disruptions in synaptogenesis and neural circuit formation. Long-term consequences may involve cognitive impairments, neurological disorders, and increased susceptibility to neurodegenerative diseases like Alzheimer’s and Parkinson’s. This is potentially due to mechanisms involving oxidative stress, neuroinflammation, disrupted proteostasis, and impaired mitochondrial function in neurons. Additionally, these compounds have been associated with altered levels of neurotransmitters such as dopamine, serotonin, and acetylcholine, leading to disruptions in synaptic transmission, neuronal signaling, and overall brain function, contributing to cognitive and behavioral deficits [81].

The potential mechanisms of neurotoxicity for TCIPP and TCEP include altered neurotransmitter levels, oxidative stress and neural damage, disruption of neural signalling pathways, endocrine disruption, neuroinflammation and glial cell activation, and mitochondrial dysfunction and energy impairment. These compounds may interfere with the synthesis, release, reuptake, or metabolism of neurotransmitters, leading to imbalances in their levels and disruptions in neural communication [3,82,83,84]. They have also been shown to induce oxidative stress in various cell types, including neurons, by generating reactive oxygen species (ROS) and depleting antioxidant defenses, resulting in oxidative damage to lipids, proteins, and nucleic acids, which can cause neuronal dysfunction, synaptic impairment, and cell death [84,85,86]. Disruptions in critical signaling pathways involved in neuronal survival, differentiation, and synaptic plasticity, such as the MAPK, PI3K/Akt, and Wnt signaling pathways, can impair neuronal development, synaptic function, and neural plasticity, contributing to cognitive and behavioral deficits [87,88]. As endocrine-disrupting chemicals, TCIPP and TCEP may interfere with hormones essential for brain development and function, such as thyroid hormones, estrogens, and androgens, potentially leading to neurodevelopmental toxicity and long-term neurological consequences. Exposure to these compounds has also been linked to activation of glial cells (astrocytes and microglia) in the brain, resulting in neuroinflammatory responses that contribute to neuronal damage, synaptic dysfunction, and neurodegeneration. Mitochondrial dysfunction, leading to disrupted energy production and increased oxidative stress, can exacerbate neurodegenerative processes through neuronal dysfunction, synaptic impairment, and cell death [89,90].

While most studies have focused on the effects of TCIPP and TCEP on the central nervous system, emerging evidence suggests potential impacts on the peripheral nervous system as well [8,28]. Neurotoxicity in dorsal root ganglia (DRG) neurons, responsible for transmitting sensory information from the periphery to the spinal cord and brain, has been reported, with proposed mechanisms including oxidative stress, mitochondrial dysfunction, and disruption of calcium homeostasis, potentially leading to sensory neuropathies or alterations in pain perception. These compounds have also been shown to interfere with neuromuscular junction function, potentially affecting muscle contraction and movement coordination, through mechanisms such as disruption of acetylcholine release and signaling, as well as interference with calcium homeostasis and muscle excitation-contraction coupling [3,83,84,91]. Additionally, some studies suggest that exposure to TCIPP and TCEP may contribute to the development of peripheral neuropathies, characterized by damage to peripheral nerves and symptoms like numbness, tingling, and muscle weakness, through mechanisms including oxidative stress, inflammation, and disruption of axonal transport and nerve conduction [56,81,92,93].

It is important to note that the neurotoxic effects of TCIPP and TCEP can vary depending on factors such as exposure levels, duration, timing, individual susceptibilities, and potential interactions with other environmental or genetic factors [81,93]. Ongoing research efforts aim to further elucidate the neurotoxic mechanisms of TCIPP and TCEP and assess their potential impact on human health. Key areas of ongoing research include epidemiological studies to assess associations between exposure to these compounds and neurological disorders in human populations, biomonitoring studies to measure their levels in biological samples and correlate them with neurotoxic outcomes, and mechanistic studies utilizing advanced in vitro and in vivo models and omics technologies to identify gene expression changes, protein modifications, and metabolic alterations associated with neurotoxicity [94,95,96,97]. Developmental neurotoxicity studies aim to assess the potential neurodevelopmental effects of TCIPP and TCEP exposure during critical windows and investigate long-term consequences on cognitive functions, behavior, and susceptibility to neurodevelopmental and neurodegenerative diseases [98]. Additionally, efforts to explore potential therapeutic interventions or preventive measures to mitigate the neurotoxic effects of TCIPP and TCEP, such as antioxidants or anti-inflammatory agents, and the development of safer alternatives with reduced neurotoxic potential are ongoing.

Regulatory and risk assessment efforts continuously review and update guidelines and exposure limits for these compounds based on the latest scientific evidence on their neurotoxic potential, incorporating neurotoxicity data into risk assessment models, and developing appropriate risk communication strategies to inform the public and policymakers about potential neurological risks associated with exposure to these flame retardants. Addressing the neurotoxic potential of TCIPP and TCEP requires a multidisciplinary approach involving experts from various fields, including neuroscience, toxicology, epidemiology, and regulatory science, to integrate data from various research approaches and foster collaborations among stakeholders, ultimately leading to the development of effective risk management strategies and the protection of human health. While the focus has been on TCIPP and TCEP, the broader class of organophosphate flame retardants (OPFRs) raises similar concerns regarding potential neurotoxicity [95,96,97,99]. As research continues to unravel the mechanisms and effects of these compounds, it may shed light on the neurotoxic potential of other OPFRs and inform the development of safer alternatives for the entire class of flame retardants.

## 7. Other Potential Toxicities

In addition to the well-studied toxic effects of TCIPP and TCEP on various systems, such as oxidative stress, endocrine disruption, genotoxicity, and neurotoxicity, these flame retardants have also been associated with other potential toxicities, including hepatotoxicity, nephrotoxicity, cardiotoxicity, developmental and reproductive toxicity, and immunotoxicity. (Table 6). Understanding these additional toxicities is crucial for comprehensively evaluating the potential health risks associated with exposure to TCIPP and TCEP. Hepatotoxicity has been evidenced by several studies reporting liver injury and dysfunction in experimental animal models exposed to these compounds. Observed effects include increased liver enzyme levels (e.g., ALT and AST), indicating hepatocellular damage, and histopathological changes such as hepatocyte necrosis, inflammation, and fatty liver disease. The mechanisms underlying hepatotoxicity involve oxidative stress and ROS generation in hepatocytes, interference with mitochondrial function, disruption of cellular signaling pathways, and induction of endoplasmic reticulum stress, along with potential interactions with hepatic metabolizing enzymes and transporters. Nephrotoxicity has also been demonstrated, with exposure leading to kidney injury and impaired renal function, as evidenced by increased levels of biomarkers such as BUN and creatinine, and histopathological changes like tubular necrosis, glomerular damage, and interstitial fibrosis. The nephrotoxic mechanisms include oxidative stress, interference with renal transporters, disruption of cellular signaling, and induction of apoptosis in renal cells (Table 6). Cardiotoxicity from TCIPP and TCEP exposure includes altered cardiac function, structural heart changes, and an increased risk of cardiovascular diseases. Effects observed are arrhythmias, changes in blood pressure, cardiac hypertrophy, and vascular endothelial dysfunction. The mechanisms involve oxidative stress, interference with ion channels and calcium homeostasis in cardiomyocytes, and disruption of hormonal signaling pathways.

Developmental and reproductive toxicity occurs when exposure happens during critical developmental periods, leading to structural malformations, growth retardation, neurodevelopmental deficits, and increased susceptibility to other toxic insults. In experimental animals, reproductive toxicity has manifested as reduced sperm count and motility, testicular atrophy in males, and ovarian function disruptions and adverse pregnancy outcomes in females. Mechanisms include endocrine disruption, oxidative stress, DNA damage, and epigenetic modifications. Immunotoxicity involves changes in immune cell populations, alterations in cytokine and immunoglobulin levels, and impaired immune responses [56,70,100,101,102] (Table 6).

Mechanisms involve oxidative stress-induced damage to immune cells, interference with immune cell function, and hormonal signaling disruption. These immunotoxic effects can increase susceptibility to infections, autoimmune disorders, and immune-related diseases, potentially compromising vaccine efficacy [103]. The severity and specific manifestations of these toxicities can vary depending on exposure levels, duration, timing, and individual susceptibilities, with co-exposure to other environmental contaminants or lifestyle factors potentially modulating the effects [104]. Ongoing research efforts aim to further elucidate the mechanisms underlying these toxicities and assess their potential impacts on human health through epidemiological studies and biomonitoring efforts, which is crucial for informing risk assessment, regulatory decisions, and the development of safer alternatives. Moreover, while the focus has been on TCIPP and TCEP, other organophosphate flame retardants (OPFRs) may share similar toxicological profiles, highlighting the broader implications for public health and the need for safer alternatives for the entire class of flame retardants.

## 8. Conclusions

In conclusion, TCIPP and TCEP are widely used flame retardants with diverse toxicities that pose significant risks to human health and the environment. Current research highlights mechanisms involving oxidative stress, inflammation, endocrine disruption, genotoxicity, and neurotoxicity, with potential for hepatotoxicity, nephrotoxicity, cardiotoxicity, developmental/reproductive toxicity, and immunotoxicity. The wide range of toxic effects, impacting multiple organ systems, necessitates a holistic and systematic research strategy.

Future research prospects should focus on unraveling the intricate molecular dynamics of these toxicants, utilizing advanced technologies such as omics and computational modeling to gain deeper insights into their mechanisms of action. Interdisciplinary studies are crucial to comprehensively understand the interactions between these compounds and biological systems.

Effective protection from the toxic effects of TCIPP and TCEP requires a multi-faceted approach that includes enhanced risk assessment models incorporating the latest mechanistic insights, as well as stricter regulations to limit the use of hazardous flame retardants. Additionally, there is a need for the development of safer alternatives to replace TCIPP and TCEP in various applications.

Reducing human exposure, particularly among vulnerable populations such as children and pregnant women, is essential. This can be achieved through improved product labeling, raising public awareness, and advocating for safer product choices. Environmental protection efforts, including enhanced monitoring and remediation, are also vital to reduce the release and persistence of these compounds in the ecosystem.

Addressing this complex toxicological issue requires collaboration among scientists, regulators, industry stakeholders, and the public. By integrating advanced research, effective regulation, and proactive measures to reduce exposure, we can better protect human health and the environment, paving the way for a healthier and more sustainable future.

## Figures and Tables

**Table 1:** Summary of ADME (Absorption, Distribution, Metabolism, and Excretion) Characteristics and Potential Toxic Effects of TCIPP and TCEP.

ADME Process	TCIPP	TCEP	Key Points/Factors Influencing
Absorption			
Routes of Exposure	Inhalation, ingestion, dermal exposure	Inhalation, ingestion, dermal exposure	Inhalation significant in occupational and indoor environments; ingestion from contaminated food, water, or dust; dermal influenced by concentration, formulation, exposure duration
Distribution			
Systemic Circulation	Yes	Yes	Both compounds distribute via systemic circulation
Tissue Accumulation	Blood, urine, breast milk, adipose tissue	Blood, urine, breast milk, adipose tissue	Lipophilicity, protein binding, metabolic processes influence distribution
Metabolism			
Primary Site	Liver	Liver	Metabolism primarily occurs in the liver
Enzymes Involved	CYP3A4, CYP2B6, CYP2E1; UGTs, SULTs, GSTs	CYP3A4, CYP2B6, CYP2E1; UGTs, SULTs, GSTs	Phase I and Phase II reactions catalyzed by various enzymes
Metabolic Reactions	Hydroxylation, dehalogenation, dechlorination, conjugation with glucuronic acid, sulfate, or glutathione	Hydroxylation, dehalogenation, dechlorination, conjugation with glucuronic acid, sulfate, or glutathione	Formation of metabolites with varying toxicological properties
Excretion			
Routes of Excretion	Urine, feces	Urine, feces	Urinary excretion significant for both compounds
Detected Metabolites	Hydroxylated, conjugated forms in urine	Hydroxylated, conjugated forms in urine	Rate of excretion influenced by metabolic biotransformation, protein binding, renal and hepatic function
Potential Toxic Effects			
Mechanisms	Oxidative stress, covalent binding, glutathione depletion, endocrine disruption, mitochondrial dysfunction, immunotoxicity	Oxidative stress, covalent binding, glutathione depletion, endocrine disruption, mitochondrial dysfunction, immunotoxicity	Influenced by genetic variability, co-exposure to other substances, age, sex, health status

This table provides an organized overview of the ADME characteristics and potential toxic effects of TCIPP and TCEP.

**Table 2:** Oxidative Stress and Inflammatory Responses Induced by TCIPP and TCEP.

Category	TCIPP	TCEP	Key Points/Factors Influencing
Oxidative Stress			
ROS Generation	Direct ROS generation through redox cycling; disruption of redox homeostasis; mitochondrial dysfunction	Direct ROS generation through redox cycling; disruption of redox homeostasis; mitochondrial dysfunction	Involves superoxide radicals, hydrogen peroxide, hydroxyl radicals; mitochondrial electron transport chain interference
Glutathione Depletion	Conjugation with metabolites depletes cellular antioxidant reserves	Conjugation with metabolites depletes cellular antioxidant reserves	Reduces ability to neutralize ROS; upregulates NADPH oxidases
Cellular Damage	DNA damage, lipid peroxidation, protein oxidation	DNA damage, lipid peroxidation, protein oxidation	Leads to genotoxicity, mutagenesis, carcinogenesis, structural/functional alterations in cell membranes, enzyme inactivation
Disruption of Signalling Pathways	Affects cell proliferation, differentiation, survival	Affects cell proliferation, differentiation, survival	Redox-sensitive pathways; potential tissue damage
Inflammatory Responses			
Pathway Activation	NF-κB, MAPK (p38, JNK)	NF-κB, MAPK (p38, JNK)	Regulates pro-inflammatory mediators
Pro-inflammatory Mediators	Increased IL-6, TNF-α, IL-1β production	Increased IL-6, TNF-α, IL-1β production	Promotes inflammation, acute phase proteins, immune cell recruitment
COX-2 Expression	Increased expression	Increased expression	Synthesis of pro-inflammatory prostaglandins
Chemokines Production	IL-8, MCP-1	IL-8, MCP-1	Chemotaxis and recruitment of immune cells to inflammation sites
Reactive Nitrogen Species	Generation of nitric oxide, peroxynitrite	Generation of nitric oxide, peroxynitrite	Exacerbates oxidative stress, cellular damage
Toxic Effects and Pathological Conditions			
Genotoxicity and Carcinogenesis	DNA damage, inflammation-mediated genomic instability	DNA damage, inflammation-mediated genomic instability	Increased risk of mutagenesis and carcinogenesis
Reproductive and Developmental Toxicity	Disrupts reproductive function, embryonic development	Disrupts reproductive function, embryonic development	Adverse reproductive and developmental outcomes
Neurotoxicity	Oxidative stress, neuroinflammation	Oxidative stress, neuroinflammation	Neuronal damage, impaired cognitive function, neurodegenerative processes
Metabolic Disorders	Insulin resistance, obesity, dyslipidemia	Insulin resistance, obesity, dyslipidemia	Chronic inflammation, oxidative stress
Cardiovascular Disease	Endothelial dysfunction, atherosclerosis	Endothelial dysfunction, atherosclerosis	Promotes cardiovascular disease
Organ Dysfunction	Liver, kidney tissue injury	Liver, kidney tissue injury	Excessive ROS production, inflammatory responses
Influencing Factors	Exposure duration, dose, individual susceptibility, co-exposures	Exposure duration, dose, individual susceptibility, co-exposures	Variability in severity and specific manifestations

This table provides a detailed summary of the oxidative stress and inflammatory responses induced by TCIPP and TCEP, including the mechanisms of ROS generation, glutathione depletion, cellular damage, pathway activation, and the resulting toxic effects and pathological conditions. The information highlights key points and factors influencing these processes.

**Table 3:** Endocrine-Disrupting Effects of TCIPP and TCEP.

Hormone Receptors and Signalling Pathways	TCIPP	TCEP	Key Points
Estrogen Receptors (ERs)	Estrogenic and anti-estrogenic activities; modulation of transcriptional activity; changes in estrogen-responsive gene expression	Estrogenic and anti-estrogenic activities; modulation of transcriptional activity; changes in estrogen-responsive gene expression	Impacts reproductive function, bone health, cardiovascular system
Androgen Receptors (ARs)	Anti-androgenic activities; disruption of androgen-mediated signalling pathways; gene expression modulation	Anti-androgenic activities; disruption of androgen-mediated signalling pathways; gene expression modulation	Essential for male sexual development, reproductive function, metabolic processes
Thyroid Hormone Homeostasis	Interference with synthesis, transport, metabolism of thyroid hormones; alterations in circulating levels; disruption of signalling pathways	Interference with synthesis, transport, metabolism of thyroid hormones; alterations in circulating levels; disruption of signalling pathways	Crucial for growth, development, metabolic regulation
Other Nuclear Receptors (PPARs)	Interaction potential; disruption of lipid, glucose metabolism; inflammatory responses	Interaction potential; disruption of lipid, glucose metabolism; inflammatory responses	Involved in metabolic homeostasis, potential for metabolic disorders

The table above gives a summary of TCIPP and TCEP’s endocrine disruption actions; these are in relation to their interaction with hormone receptors and signal transduction pathways. Some important aspects include the potential effects on reproductive systems, development, metabolic processes, as well as their associated adverse impacts.

**Table 4:** Genotoxic and Carcinogenic Potential of TCIPP and TCEP.

Aspect	TCIPP	TCEP	Key Points
Genotoxicity Evaluation			
In vitro Studies	Negative or weakly positive results in bacterial mutagenicity assays; mixed results in mammalian cell genotoxicity assays	Negative or weakly positive results in bacterial mutagenicity assays; mixed results in mammalian cell genotoxicity assays	Limited gene mutation potential in bacteria; positive findings for chromosomal aberrations, micronucleus formation, DNA strand breaks in mammalian cells
In vivo Studies	Mixed results in chromosomal aberration assays; positive findings in some micronucleus assays; positive results in comet assays	Mixed results in chromosomal aberration assays; positive findings in some micronucleus assays; positive results in comet assays	Chromosomal damage, aneuploidy, DNA strand breaks observed
Mechanisms of DNA Damage			
Oxidative Stress	Induction of reactive oxygen species (ROS) leading to oxidative DNA damage	Induction of reactive oxygen species (ROS) leading to oxidative DNA damage	Causes various DNA lesions, including oxidized bases, single- and double-strand breaks
Direct DNA Interaction	Potential covalent binding or intercalation with DNA; DNA adduct formation	Potential covalent binding or intercalation with DNA; DNA adduct formation	Interferes with DNA replication, transcription, repair processes
DNA Repair Mechanisms Inhibition	Inhibition of DNA repair enzymes (DNA glycosylases, topoisomerases)	Inhibition of DNA repair enzymes (DNA glycosylases, topoisomerases)	Impairs genomic integrity maintenance, increases mutagenesis risk
Epigenetic Modifications	Potential induction of DNA methylation changes, histone modifications	Potential induction of DNA methylation changes, histone modifications	Alters gene expression patterns, contributes to carcinogenesis
Carcinogenic Classification by Regulatory Agencies	Group 3 (“Not classifiable”) by IARC for TCIPP; Group 2A (“Probably carcinogenic”) for TCEP; “Suggestive Evidence of Carcinogenic Potential” for TCIPP by EPA; “Likely to Be Carcinogenic” for TCEP by EPA; Suspected carcinogens (Carc. 2) by ECHA for both TCIPP and TCEP	Group 3 (“Not classifiable”) by IARC for TCIPP; Group 2A (“Probably carcinogenic”) for TCEP; “Suggestive Evidence of Carcinogenic Potential” for TCIPP by EPA; “Likely to Be Carcinogenic” for TCEP by EPA; Suspected carcinogens (Carc. 2) by ECHA for both TCIPP and TCEP	Various classifications based on evidence of mutagenicity, carcinogenicity in animals, structural activity relationships
Potential Carcinogenic Mechanisms	DNA damage initiation; tumor promotion via cellular proliferation, apoptosis inhibition, pro-inflammatory environment; potential hormone-related cancer promotion	DNA damage initiation; tumor promotion via cellular proliferation, apoptosis inhibition, pro-inflammatory environment; potential hormone-related cancer promotion	Genotoxic effects initiate carcinogenesis; cellular processes support tumor growth and survival

**Table 5:** Neurotoxic Effects of TCIPP and TCEP.

Neurotoxic Mechanisms	Potential Impact on Nervous System	Key Findings
Altered Neurotransmitter Levels	Disruptions in synaptic transmission, neural signalling, and overall brain function	Imbalances in neurotransmitters (dopamine, serotonin, acetylcholine) lead to cognitive and behavioral deficits
Oxidative Stress and Neural Damage	Neuronal dysfunction, synaptic impairment, cell death	Generation of reactive oxygen species (ROS) leads to oxidative damage to lipids, proteins, and nucleic acids
Disruption of Neural Signalling Pathways	Impaired neuronal survival, synaptic function, neural plasticity	Interference with critical signalling pathways (MAPK, PI3K/Akt, Wnt) affects cognitive functions and behavior
Endocrine Disruption	Neurodevelopmental toxicity, long-term neurological consequences	Interference with essential hormones for brain development and function
Neuroinflammation and Glial Cell Activation	Neuronal damage, synaptic dysfunction, neurodegeneration	Activation of astrocytes and microglia leads to inflammatory responses
Mitochondrial Dysfunction and Energy Impairment	Neuronal dysfunction, synaptic impairment, cell death	Disruption of energy production and increased oxidative stress
Peripheral Nervous System Effects	Sensory neuropathies, alterations in pain perception	Reported neurotoxicity in dorsal root ganglia (DRG) neurons
	Neuromuscular junction dysfunction, muscle contraction issues	Interference with acetylcholine release, calcium homeostasis, muscle excitation-contraction coupling
	Peripheral neuropathies, numbness, tingling, muscle weakness	Damage to peripheral nerves, disruption of axonal transport and nerve conduction

**Table 6:** Additional Toxicities Associated with TCIPP and TCEP.

Toxicity	Observed Effects	Mechanisms
Hepatotoxicity	Liver injury, increased liver enzyme levels (ALT, AST), histopathological changes (hepatocyte necrosis, inflammation)	Oxidative stress, interference with mitochondrial function, disruption of cellular signalling pathways, induction of endoplasmic reticulum stress
Nephrotoxicity	Kidney injury, increased biomarker levels (BUN, creatinine), histopathological changes (tubular necrosis, fibrosis)	Oxidative stress, interference with renal transporters, disruption of cellular signalling pathways, induction of apoptosis in renal cells
Cardiotoxicity	Alterations in cardiac function, structural changes in the heart, increased risk of cardiovascular diseases	Oxidative stress, interference with ion channels and calcium homeostasis, disruption of hormonal signalling pathways involved in cardiovascular function
Developmental Toxicity	Structural malformations, growth retardation, neurodevelopmental deficits	Adverse outcomes during critical developmental periods, endocrine disruption, oxidative stress, DNA damage, epigenetic modifications
Reproductive Toxicity	Reduced sperm count and motility, testicular atrophy, disruptions in ovarian function, adverse pregnancy outcomes	Endocrine disruption, oxidative stress, DNA damage, epigenetic modifications
Immunotoxicity	Alterations in immune cell populations, changes in cytokine levels, impaired immune responses	Oxidative stress-induced damage to immune cells, interference with immune cell function, disruption of hormonal signalling pathways

## Data Availability

Data is available with in the article.
